# Work-based Assessment and Co-production in Postgraduate Medical Training

**DOI:** 10.3205/zma001135

**Published:** 2017-11-15

**Authors:** Eric S. Holmboe

**Affiliations:** 1 Accreditation Council for Graduate Medical Education, Chicago, USA

**Keywords:** competency-based medical education models, Co-production of assessment, programmatic assessment

## Abstract

Assessment has always been an essential component of postgraduate medical education and for many years focused predominantly on various types of examinations. While examinations of medical knowledge and more recently of clinical skills with standardized patients can assess learner capability in controlled settings and provide a level of assurance for the public, persistent and growing concerns regarding quality of care and patient safety worldwide has raised the importance and need for better work-based assessments. Work-based assessments, when done effectively, can more authentically capture the abilities of learners to actually provide safe, effective, patient-centered care. Furthermore, we have entered the era of interprofessional care where effective teamwork among multiple health care professionals is now paramount. Work-based assessment methods are now essential in an interprofessional healthcare world.

To better prepare learners for these newer competencies and the ever-growing complexity of healthcare, many post-graduate medical education systems across the globe have turned to outcomes-based models of education, codified through competency frameworks. This commentary provides a brief overview on key methods of work-based assessment such as direct observation, multisource feedback, patient experience surveys and performance measures that are needed in a competency-based world that places a premium on educational and clinical outcomes. However, the full potential of work-based assessments will only be realized if post-graduate learners play an active role in their own assessment program. This will require a substantial culture change, and culture change only occurs through actions and changed behaviors. Co-production offers a practical and philosophical approach to engaging postgraduate learners to be active, intrinsically motivated agents for their own professional development, help to change learning culture and contribute to improving programmatic assessment in post-graduate training.

## Zusammenfassung

Prüfungen waren schon immer ein wesentlicher Bestandteil der postgraduierten medizinischen Weiterbildung und haben sich über viele Jahre vorwiegend auf verschiedene Prüfungsformen konzentriert. Durch die Prüfung medizinischen Wissens und neuerdings klinischer Fertigkeiten mit Hilfe von standardisierten Patienten können die Leistungen des Lernenden unter kontrollierten Bedingungen bewertet und eine gewisse Qualitätssicherung für die Öffentlichkeit gewährleistet werden. Anhaltende und wachsende Bedenken bezüglich der Versorgungsqualität und Patientensicherheit weltweit haben die Bedeutung und Notwendigkeit von besseren arbeitsbasierten Bewertungen erhöht. Effektiv durchgeführt können diese die Fähigkeit der Lernenden, eine sichere, wirksame und patientenorientierte Versorgung durchzuführen, verlässlicher erfassen. Außerdem befinden wir uns in einem Zeitalter der interprofessionellen medizinischen Versorgung, in der effektive Teamarbeit zwischen verschiedenen Gesundheitsberufen ganz im Vordergrund steht. Arbeitsbasierte Bewertungsmethoden sind in einer interprofessionellen Welt der Gesundheitsversorgung unverzichtbar.

Um Lernende besser auf diese neuen Kompetenzen und die zunehmende Komplexität der Gesundheitsversorgung vorzubereiten, haben viele postgraduierte medizinische Weiterbildungssysteme weltweit Outcome-basierte Weiterbildungsmodelle aufgegriffen, die sich an kompetenzorientierten Rahmenstrukturen orientieren. Dieser Artikel bietet einen kurzen Überblick über Schlüsselmethoden der arbeitsbasierten Bewertung, wie etwa die direkte Beobachtung, Multisource-Feedback, Patientenerfahrungsumfragen und Leistungsbewertungen, die in einer kompetenzbasierten Weiterbildung erforderlich sind, in der klinischen und bildungsorientierten Ergebnissen (Outcomes) ein besonderer Wert zukommt. Allerdings wird das volle Potenzial arbeitsbasierter Bewertungen erst dann ausgeschöpft, wenn postgraduierte Lernende selbst eine aktive Rolle in ihrem Prüfungsprogramm übernehmen. Voraussetzung ist ein entscheidender Kulturwandel, der nur durch aktives Handeln und eine Verhaltensänderung eintreten kann. Das Modell der Co-Produktion bietet eine praktische und philosophische Herangehensweise, um postgraduierte Lernende für eine aktive und intrinsisch motivierte Partizipation an ihrer eigenen professionellen Entwicklung zu gewinnen, dadurch die Lernkultur zu verändern und zur Verbesserung eines programmatischen Prüfungskonzeptes für die postgraduierte Weiterbildung beizutragen.

## Background

Assessment has always been an essential aspect of learning across all health professions. Assessment *of* learning, or more specifically of knowledge, dominated medical education in the last century [1]. Toward the latter quarter of the 20^th^ century important advances were made in simulation to the point approaches such as the objective structured clinical examinations (OSCEs) became a core component of some national licensing processes [2]. In 1990 George Miller published his now iconic assessment pyramid highlighting the levels of knows, knows how, shows how and does [3]. Assessments such as knowledge examinations and OSCEs address the knows, knows how and shows how levels and have been important in assuring the public a minimal level of competence among health professionals.

However, as the use of examinations and OSCEs expanded evidence of major problems and deficiencies were also emerging around the delivery of health care. Over the last 40 plus years multiple studies have demonstrated substantial issues in patient safety, medical errors, underuse of evidence-based therapies, lack of patient-centeredness and overuse of tests, procedures and therapies that have limited to no medical value [4], [5], [6], [7]. For example, the Commonwealth Fund routinely publishes comparative data from multiple countries that show all heath systems have substantial room to improve [8]. Recently, Makary and Daniel found medical errors may be the third leading cause of death in the United States [9].

It was against this backdrop of quality and safety concerns that the outcomes-based education movement began to take hold in a number of countries [10], [11], [12], [13]. Medical education began to shift from a process model (i.e. simply completing a course of instruction and experiential activities) to an outcomes model focused on determining what graduates can actually do when caring for patients and families. This trend became particularly evident in post-graduate training programs where the predominant form of learning is experiential by caring for patients and families. Educational outcomes in postgraduate training were subsequently codified as competencies [12], [13]. A competency is simply an observable ability of a health professional, integrating multiple components such as knowledge, skills, values and attitudes [14]. Competencies provide the framework for curricula and assessment to ensure health professional can perform the key clinical activities of their discipline.

The rapidly increasing complexity of both basic medical and healthcare delivery sciences mandated a reexamination of what abilities were needed by professionals for modern and future practice. In the United States, specialty postgraduate training, called residencies, typically occurs after four years of college subsequently followed by four years of medical school. Residency education in the United States has been guided by six general, or domains of, competencies since 2001: patient care, medical knowledge, professionalism, interpersonal skills and communication, practice based learning and improvement and systems-based practice [13]. The competencies of practice-based learning and improvement and systems-based practice were specifically created to target areas such as evidence-based practice and use of clinical decision support systems and resources, quality improvement, patient safety science, care coordination, interprofessional teamwork, effective use of health information technology and stewardship of resources. Greater attention was also directed toward communication skills such as shared and informed decision making, health literacy and numeracy. 

It became increasingly clear that traditional methods of assessment that targeted the knows, knows how and show how levels were insufficient to assess these critical new competencies and meet the needs of patients [15], [16]. Furthermore, the consequences from decades of not adequately observing and assessing the core clinical skills of medical interviewing, physical examination, informed decision making and clinical reasoning have had a major negative impact on quality of care [17], [18]. This was also part of George Miller’s message of the importance of the does level of the pyramid, determining what health professionals can actually do is the ultimate goal of education and assessment [3]. 

Competencies have also been difficult to implement. Faculty often weight competencies idiosyncratically, too many assessment tools presumably designed for a competency-based approach are overly reductionist (e.g. a tick box exercise) and there has been a general lack of a programmatic approach to assessment [19], [20]. Finally, culture and the factors that enable learner engagement are also beginning to emerge that will have a major impact on how learners embrace assessment as active participants [21], [22]. These factors, among others, have led to the push for more and better work-based assessments. 

## Emergence of Work-based Assessment

While work-based assessments (WBAs) have been used for a long time, they have been, and in many cases still are, used poorly and ineffectively [17]. One example is the faculty evaluation form typically completed at the end of a clinical experience or rotation. Multiple past studies show rating problems such as range restriction, halo and leniency effects accompanied by few to no narrative comments for feedback [16], [23]. Much of the ratings were and are often still based on proxies such as case presentations and rounds away from the bedside with little direct observation of the learner with patients and families. This lack of effective assessment can also lead to the “failure to fail” phenomenon where residents are promoted despite possessing significant dyscompetencies that potentially leads to patient harm [24].

**Figure 1:**
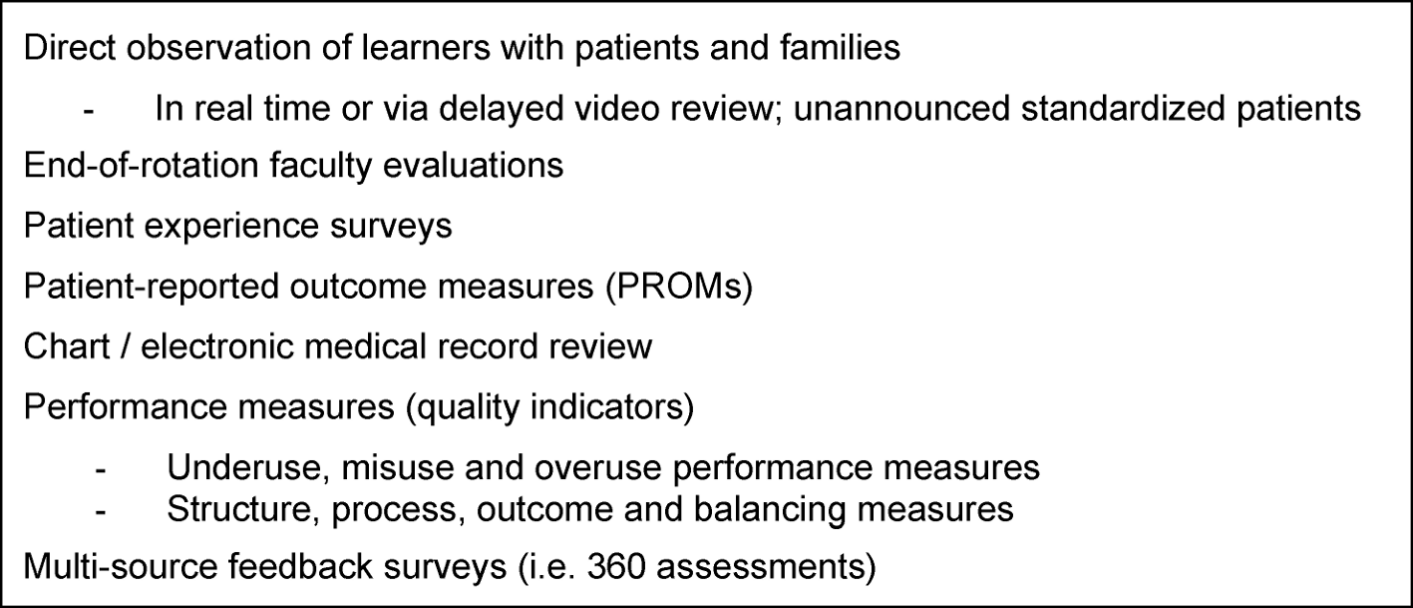
Examples of Work-based Assessments

However, the last 15 years has seen the emergence of a number of new work-based assessment tools and approaches (see figure 1 (Fig. 1)). First, research on rater cognition have deepened our understanding on what affects faculty observations along with promising techniques to help faculty perform observation more effectively [25]. For example, Kogan and colleagues found using interactive, conversational methods to deepen understanding of key clinical competencies, known as performance dimension and frame of reference training, can actually empower faculty in their observations of medical residents and may actually transfer to improving their own personal clinical skills [26]. While much research and evaluation remains to be done, research is beginning to provide helpful guidance on how to better prepare faculty to assess through direct observation.

Another important development is the use of construct-aligned scales, especially using entrustment type anchors involving either developmental stages and/or supervision. For example, Crossly and colleagues found using scales focused on the constructs of “developing clinical sophistication and independence, or ‘entrustability’” in three assessment tools for the United Kingdom Foundation program led to better reliability and higher satisfaction among the faculty using these tools [27]. Weller and colleagues found similar effects in anesthesia training when using an entrustment supervision scale [28].

Multisource feedback (MSF), including the increasing use of patient experience surveys, is another important development in work-based assessment [29]. Feedback from peers, patients, families and other members of the healthcare team can be useful and powerful aids to professional development and is especially important in reconfigured healthcare delivery that recognizes the critical importance of interprofessional teamwork to deliver safe, effective patient-centered care. Another growing area is the use of patient reported outcome measures (PROMs) that specifically target functional outcomes for patients [30]. For example, while avoiding deep venous thrombosis and infection is paramount in the peri-operative period after joint arthroplasty, the ultimate goal is to improve physical function. The Oxford hip and knee scales are examples of PROMs currently in use and can be useful adjuncts to a program of assessments [30].

## Performance Measures in Postgraduate Education

Performance measures *“are designed to measure systems of care and are derived from clinical or practice guidelines. Data that are defined into specific measurable elements provides an organization with a meter to measure the quality of its care.*” [31]. Performance measures typically fall into four main categories (see Table 1 (Tab. 1)) [32], [31]. In addition to patient experience measures, measurement and assessment of clinical performance is now an essential component of both quality assurance and improvement efforts and will need to increasingly find their way into health professions training. One method to assess clinical performance in addition to direct observation, patient surveys and MSF is review, or audit, of the medical record [33]. Medical records serve a number of important functions:

1. as an archive of important patient medical information for use by other healthcare providers and patients;
2. as source of data to assess performance in practice such as chronic medical conditions (e.g. diabetes), post-operative care or prevention;
3. monitoring of patient safety and complications; and
4. documentation of diagnostic and therapeutic decisions.

One can readily see how the medical record can be used for educational and assessment purposes [33], [34], [35], [36], [37]. 

For health professions training programs, the first three types of measures are important to examine the relationships between the quality of care provided and educational outcomes and ensure the learning environment is performing optimally. For more advanced training programs, additional experience in balanced measures (“balanced scorecards”) [31] may be useful. For the individual learner, process and outcome measures will usually be the most useful types of measures for assessment, feedback and continuous professional development.

Practice reviews are an essential method in the evaluation of the competencies of *Practice-based Learning and Improvement* (PBLI) and *Systems-based Practice* (SBP) in the United States general competency framework [13] and the *Leader* role in CanMEDS [38]. These competencies require that residents be actively involved in monitoring their own clinical practice and improving the quality of care based on a systematic review of the care they provide. Practice review, using the medical record, can promote self-reflection and support self-regulated learning, important skills needed for life-long learning. The key message here is review of clinical performance is something that learners can potentially do as a self-directed activity. 

## New Concepts to Guide Assessments

**Figure 2:**
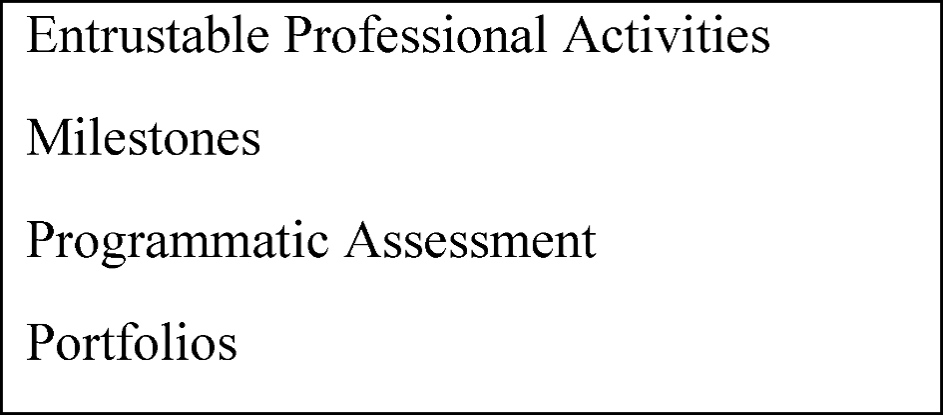
Important Concepts in Work-based Assessment

While the above work-based assessments are improving and being increasingly used, training programs have struggled to synthesize and integrate all this assessment data into judgments about learner progress. Four major developments have occurred as attempts to guide both curriculum and assessment (see figure 2 (Fig. 2)). The first newer concept is entrustable professional activities (EPAs) [39], [40], [41]. As defined by ten Cate, an EPA *“can be defined as a unit of professional practice that can be fully entrusted to a trainee, as soon as he or she has demonstrated the necessary competence to execute this activity unsupervised*.” [39], [40], [41]. The second new concept is the use of milestones to describe developmental stages of individual competencies in narrative terms [42], [43]. EPAs describe the actual activities health professionals do while milestones describe the abilities needed by the individual to effectively execute the activity (i.e. EPA). EPAs and competencies, described by milestones narratives, can be nicely combined to richly define an educational trajectory and guide what assessments are needed to determine learner progression [44]. The third major development has been the push for programmatic assessment that can effectively integrate multiple assessments into decisions and judgments and that uses group process to make judgments on progress [45], [46]. The combination of EPAs, milestones and programmatic assessment all provide useful frameworks and approaches to enhance the development and effective use of work-based assessments. While not necessarily a new concept, portfolios are growing in importance as an effective way to facilitate and manage programmatic assessment and track developmental progression through EPAs and milestones [47]. Portfolios can also enhance learner engagement. However, while advances in work-based assessment methods and concepts have helped to move outcomes-based education forward, insufficient attention has been directed toward the role of the learner in assessment. 

## Activating the Learner through Co-production

In order to achieve mastery in practice, learners need specific skills in reflection and mindful practice, self-regulated learning and informed self-assessment. Reflection on practice involves a thoughtful and deliberate review of one’s past performance (e.g. a medical record review or MSF), and is often best done in conversation with a trusted peer or advisor. This is where work-based assessment can be particularly helpful as it represents authentic work and can be reviewed in a continuous and longitudinal manner.

Self-regulated learning requires that learners set specific goals, develop strategic plans, self-monitor and self-assess as they participate in their education. Self-regulated learning thereby engages learners in the three essential elements of forethought, actual performance and subsequent self-reflection [48]. Self-regulated learning also requires awareness of the educational context and recognition that how the learner responds to and actually influences that context will affect the impact of their assessments (49). Self-regulated learning also requires substantial intrinsic motivation [48], [49]. If intrinsic motivation is not a major driver for the learner, it is less likely assessment will have the intended effects. Learners should not rely on extrinsic motivators (e.g. rewards or penalties) as a successful career strategy. Incorporating the key tenets of self-regulated learning into plans is both logical and supported by educational theory [49].

Informed self-assessment requires that learners proactively seek out assessment from faculty and members of the healthcare team, perform aspects of their own assessment such as clinical performance reviews or evidence-based practice and actively engage their assessment data for professional development as part of their own accountability [50], [51]. In other words, learners need to be producers of their own learning and assessment in collaboration and partnership with faculty and the program. This is where the concept of “co-production” can be helpful in realizing the promise of outcomes-based education.

## Co-production and Work-based Assessment

The concept of co-production was originally developed in the context of public services and can be defined as “delivering* public services in an equal and reciprocal relationship between professionals, people using services, their families and their neighbours. Where activities are co-produced in this way, both services and neighbourhoods become far more effective agents of change.*” [52]. More recently Batalden and colleagues expanded this concept for healthcare that has important implications for medical education. They defined co-production as the *“interdependent work of users and professionals to design, create, develop, deliver, assess and improve the relationships and actions that contribute to the health of individuals and populations.”* [53]. In their model, professionals and patients co-produce health and healthcare through civil discourse, co-planning and co-execution within the context of supportive institutions and the larger healthcare system.

It is not long reach to see how co-production can be applied within medical education and work-based assessment. Work-based assessment through a co-production lens could be defined as the *“interdependent work of learners, faculty, health professionals and patients to design, create, develop, deliver, assess and improve the relationships and activities that contribute to the effective assessment and professional development of learners.*” [53]. Combining the key principles of self-regulated learning, informed self-assessment and co-production makes it clear that learners must be “active agents” in their own assessment program. 

Activated learners should be empowered to ask for direct observation, feedback and coaching. Activated learners also must perform some of their own assessment such as progress testing, review of their own clinical practice, pursuit and documentation of seeking answers to clinical questions through effective evidence-based practice, seeking feedback from patients and members of interprofessional care teams and developing and executing individual learning plans. Empowering residents to seek and co-produce part of their assessments is undergirded by the tenets of self-determination theory (SDT) that posits humans possess three innate psychological needs for competence, autonomy and relatedness [54]. Equally important, SDT provides insight into how residents can internalize regulation of behavior such as assessment that has often been purely driven by faculty and the program, or in other words, more external motivators. The ultimate goal to help the resident develop autonomous, self-determined activity in assessment (54). For medicine, this will require a substantial culture change, and culture change only occurs through actions and changed behaviors. 

## Conclusion

With the rise of competency-based medical education has come a greater need for effective work-based assessments that can guide professional development and help improve entrustment decision making. EPAs, milestones and programmatic assessment can help to guide the appropriate choice and development of work-based assessments to determine meaningful educational outcomes. Finally, and perhaps most importantly, learners across the educational continuum must become active agents in their program of assessment if we are to realize the full promise of competency-based medical education models. This will require a substantial culture change, and culture change only occurs through actions and changed behaviors. Co-production of assessment by learners with faculty, training programs and patients provides a useful conceptual path to change assessment culture and move competency-based medical education forward. 

## Competing interests

Dr. Holmboe works for the Accreditation Council for Graduate Medical Education and receives royalties for a textbook on assessment from Mosby-Elsevier. 

## Figures and Tables

**Table 1:**
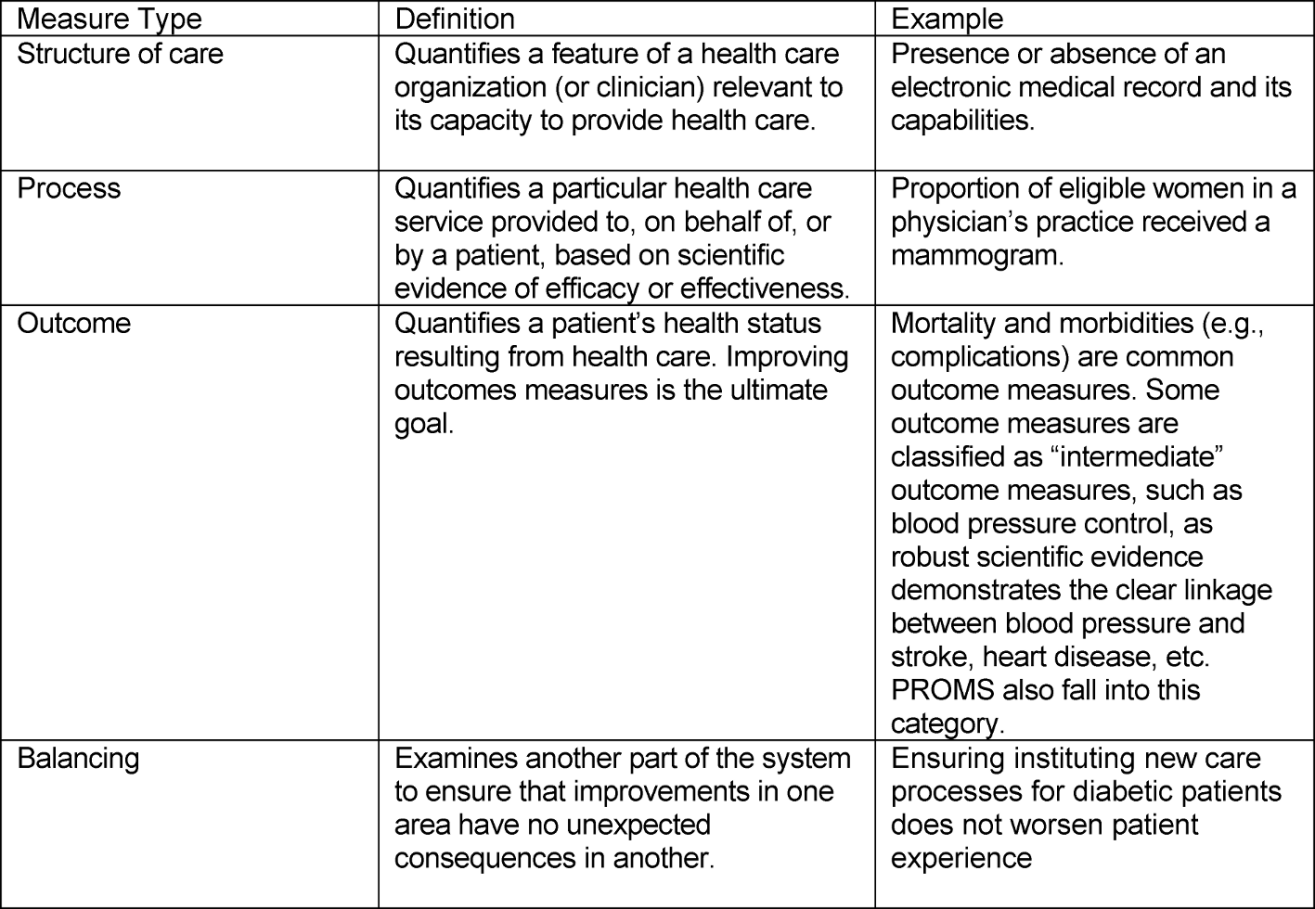
Categories and Definitions of Performance Measures
